# Prospects of Autonomous Volcanic Monitoring Stations: Experimental Investigation on Thermoelectric Generation from Fumaroles

**DOI:** 10.3390/s20123547

**Published:** 2020-06-23

**Authors:** Leyre Catalan, Miguel Araiz, Patricia Aranguren, German D. Padilla, Pedro A. Hernandez, Nemesio M. Perez, Celestino Garcia de la Noceda, Jose F. Albert, David Astrain

**Affiliations:** 1 Department of Engineering, Institute of Smart Cities, Public University of Navarre, 31006 Pamplona, Spain; miguel.araiz@unavarra.es (M.A.); patricia.arangureng@unavarra.es (P.A.); david.astrain@unavarra.es (D.A.); 2 Instituto Volcanologico de Canarias (INVOLCAN), 38320 San Cristobal de La Laguna, Spain; german@iter.es (G.D.P.); phdez@iter.es (P.A.H.); nperez@iter.es (N.M.P.); 3 Instituto Tecnologico y de Energias Renovables (ITER), 38600 Granadilla de Abona, Spain; 4 Agencia Insular de Energia de Tenerife (AIET), 38612 Granadilla de Abona, Spain; 5 Instituto Geologico y Minero de España (IGME), 28003 Madrid, Spain; c.garcia@igme.es; 6 GAIA Geotermia y Aguas Minerales S.L., 28029 Madrid, Spain; j.albert@gaiarecursos.es

**Keywords:** thermoelectric generator, geothermal, volcano, power generation, autonomous, thermoelectricity, heat pipe

## Abstract

Fumaroles represent evidence of volcanic activity, emitting steam and volcanic gases at temperatures between 70 and 100 ${}^{\circ }C$. Due to the well-known advantages of thermoelectricity, such as reliability, reduced maintenance and scalability, the present paper studies the possibilities of thermoelectric generators, devices based on solid-state physics, to directly convert fumaroles heat into electricity due to the Seebeck effect. For this purpose, a thermoelectric generator composed of two bismuth-telluride thermoelectric modules and heat pipes as heat exchangers was installed, for the first time, at Teide volcano (Canary Islands, Spain), where fumaroles arise in the surface at 82 ${}^{\circ }C$. The installed thermoelectric generator has demonstrated the feasibility of the proposed solution, leading to a compact generator with no moving parts that produces a net generation between 0.32 and 0.33 W per module given a temperature difference between the heat reservoirs encompassed in the 69–86 ${}^{\circ }C$ range. These results become interesting due to the possibilities of supplying power to the volcanic monitoring stations that measure the precursors of volcanic eruptions, making them completely autonomous. Nonetheless, in order to achieve this objective, corrosion prevention measures must be taken because the hydrogen sulfide contained in the fumaroles reacts with steam, forming sulfuric acid.

## 1. Introduction

Volcanoes are one of the most evident manifestations of geothermal energy. In active volcanoes, one way in which this geothermal energy is revealed is in the form of fumaroles, i.e., vents in the Earth’s surface from which steam and volcanic gases are emitted, normally at temperatures between 70 and 100 ${}^{\circ }C$ [1]. Monitoring these fumaroles in conjunction with other precursors is of great importance in order to predict volcanic eruptions [2,3,4]. Nevertheless, the power supply of the required equipment is a challenge due to the habitual remoteness of volcanoes.

Geothermal energy has the potential to be transformed into electricity [5], for which, traditionally, cycles have been used provided that the temperature of the geothermal field is greater than 70 ${}^{\circ }C$ [6,7]. In the low enthalpy range (70 to 150 ${}^{\circ }C$ approximately), in which fumaroles are encompassed, power is typically generated by means of binary cycles, closed cycles that convert heat from a geothermal fluid into electricity by transferring the heat to another low boiling point working fluid that drives a turbine [8]. This fluid can be an organic fluid, leading to an Organic Rankine Cycle (ORC), or ammonia, in which case the cycle is known as Kalina. Nowadays, some of the existing binary plants are already working with inlet temperatures between 70 and 100 ${}^{\circ }C$, presenting capacities up to 0.5 MW and efficiencies lower than 3% [9]. Nevertheless, binary cycles are not suitable for the considered application, since a compact, autonomous, and robust stand-alone device to supply low power is required.

One alternative in order to generate electricity from geothermal heat consists in the use of thermoelectric generators, solid-state devices that directly convert heat flux into electricity due to the Seebeck effect. For this purpose, thermoelectric generators are composed of thermoelectric modules and heat exchangers. The conversion itself takes place in the thermoelectric modules, a group of thermocouples connected electrically in series and thermally in parallel protected with ceramic sheets, while the heat exchangers are necessary in order to maximize the temperature difference between the sides of the modules, since the greater the temperature difference, the higher the generation.

Fin dissipators, liquid-based heat exchangers, heat pipes, and thermosyphons are the most common heat exchangers used in thermoelectric generators [10]. Fin dissipators stand out due to their simplicity, robustness, and low price, achieving low thermal resistances when working as active cooling systems, i.e., aided by a fan so that forced convection conditions are obtained [11,12]. On their behalf, liquid-based heat exchangers present better convection coefficients, improving the performance of the system. However, the pumps necessary to propel the liquid through the circuit require a higher auxiliary consumption and therefore reduce net generation [13,14]. Finally, heat pipes and thermosyphons are gaining attention in the last years. Making use of the latent heat of an internal fluid that cyclically vaporizes and condensates, these heat exchangers obtain low thermal resistances without requiring auxiliary equipment [15,16,17].

Thermoelectric generators present numerous advantages [18]: Direct energy conversion, avoiding the intermediate conversion of thermal energy into mechanical energy in order to generate electricity with an alternator; long lifespan, especially when working with constant reservoirs, as it has been demonstrated in spatial applications; ability to generate electricity with any temperature difference; scalability; and static and noiseless operation of the thermoelectric modules, which neither use working fluids. Nevertheless, they present an important drawback that has prevented their utilization in civil applications: Their efficiency is very low, between 2 and 5% depending on the temperature range [18,19], an efficiency very similar to the one obtained with binary plants in the temperature range considered with fumaroles.

In their application to geothermal heat, thermoelectric generators have been identified as one of the ways to speed up the installation of geothermal power [20], and therefore there exist various proposals that combine thermoelectric generators and geothermal energy. Most of them try to maximize power generation from low-medium enthalpy geothermal fields ($T<150\;{}^{\circ }C$) incorporating for this purpose liquid-based heat exchangers, similarly to their competitors, binary cycles. Some of these proposals demonstrate their feasibility by simulation, such as Suter et al., who optimized a 1 kW thermoelectric generator with a 100 ${}^{\circ }C$ temperature difference [21], or Wang et al., who proposed integrating these thermoelectric generators downhole in oil and gas wells, being able to obtain 8.5 kW in a vertical well with a 100 ${}^{\circ }C$ gradient, and 128 kW in the case of a horizontal one with a temperature difference of 156 ${}^{\circ }C$ [22,23]. In contrast, others do it with real prototypes at the laboratory, such as Liu et al. who built a 160 W thermoelectric generator composed of 96 thermoelectric modules that operated with an 80 ${}^{\circ }C$ gradient [24,25,26], or Ahiska and Mamur, who produced 41.6 W with 20 thermoelectric modules and a temperature difference of 67 ${}^{\circ }C$ [27,28], or finally, Trip et al., who, with a gradient of 72 ${}^{\circ }C$ and 40 modules, generated 0.4 W [29].

Due to the utilization of liquid-based heat exchangers, all the previous examples obtain low values of thermal resistance. However, they present an extra electrical consumption because of the pump, which reduces net generation. Catalan et al. experimentally demonstrated that passive heat exchangers based on phase change are more adequate for geothermal thermoelectric generators [30]. These heat exchangers also present low values of thermal resistance, but they do not include mobile parts nor auxiliary consumption, thus maximizing power generation and reducing maintenance requirements. While they proposed their use for a high temperature hot dry rock field, they can be extrapolated to fumaroles. In fact, Xie et al. already demonstrated the feasibility of a thermoelectric generator with a heat pipe as hot side heat exchanger in hydrothermal vents, the equivalent of fumaroles underwater, obtaining a maximum of 3.9 W with 4 thermoelectric modules from a 379 ${}^{\circ }$C vent located at a depth of 2765 m.

The objective of the present paper is to study, for the first time, the viability of thermoelectric generators in volcanic fumaroles. For this purpose, a prototype with heat exchangers based on phase change has been installed at Teide volcano. Teide is a stratovolcano located in Tenerife (Canary Islands, Spain), a volcanic island in the Atlantic Ocean whose landscape is molded by different volcanoes. Teide is not only the highest volcano on the island, with an altitude of 3718 m, but also the third highest volcano in the world from its base on the seafloor. Due to its activity, Teide volcano presents constant fumaroles at a temperature of 82 ${}^{\circ }C$, which corresponds with water vaporization temperature at that height [31,32]. These fumaroles will represent the heat source for the installed thermoelectric generator.

The interest in generating electricity from fumaroles resides in the possibility of supplying energy to the volcanic monitoring stations that aim to measure the precursors of volcanic eruptions. Most active volcanoes of the world incorporate this kind of vigilance stations, which measure different parameters such as the variation in temperature or composition of the fumaroles, or the seismic activity. The power requirements of these stations depend on the installed equipment. Nonetheless, it is normally of a few watts, with punctual peaks during communication [33,34], and with Internet of Things (IoT) technologies, it can be diminished to a few milliwatts [35]. Hence, given this low energy consumption, the proposal of thermoelectric generators with phase change heat exchangers could become the perfect energy supplier and make the stations completely autonomous: Power would be generated continuously during day and night, even improving with adverse weather conditions, the device would use passive heat exchangers that do not require auxiliary consumption nor present mobile parts, reducing maintenance requirements in locations that normally are difficult to access, and it would be very compact and easy to install.

The use of thermoelectricity for micro-generation oriented to sensors is widely available in the literature [36,37,38]. Regarding its combination with geothermal energy, two faint tendencies can be found. On the one hand, some proposals combine traditional geothermal plants with thermoelectric generators installed on the pipes to power different sensors or actuators [39,40,41]. On the other hand, others use the temperature difference between forest soil and the environment to power sensors, as proposed by Stokes et al. [42] and put into practice by Huang et al. using heat pipes as heat exchangers [43,44,45]. Nonetheless, the use of fumaroles as heat source is proposed for the first time in the present paper.

Section 2 details the thermoelectric generator installed at Teide volcano. Section 3 describes the monitoring system used. Section 4 analyzes the obtained results as well as the arisen problems. Finally, Section 5 presents the conclusions and future lines.

## 2. Thermoelectric Generator for Teide Volcano

While the most important element of a thermoelectric generator are the thermoelectric modules, heat exchangers become essential in order to maximize power generation. A reduction of 10% in the thermal resistance of the heat exchangers leads to an 8% higher generation [46]. In accordance with Catalan et al. [30], who demonstrated that heat exchangers based on phase change are the most recommended ones for geothermal thermoelectric generators, the present paper includes heat pipes at both sides of the thermoelectric modules.

**Figure 1:**
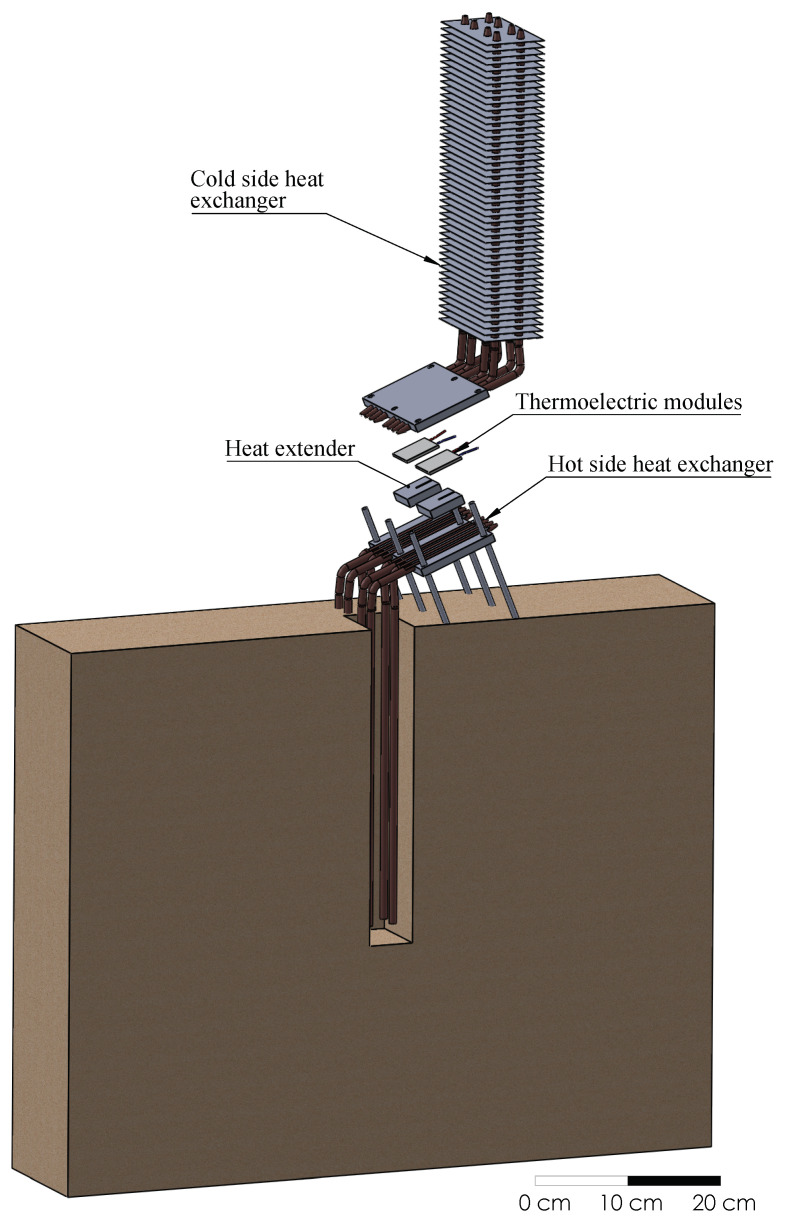
Exploded view of the geothermal thermoelectric generator installed at Teide volcano.

**Figure 2:**
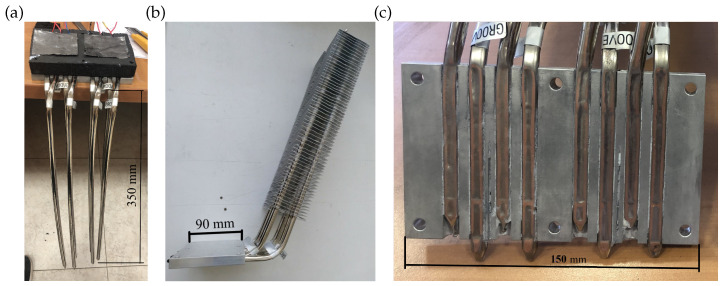
(**a**) Hot side heat exchanger. (**b**) Cold side heat exchanger. (**c**) Detail of the fitting between the heat pipe tubes and the aluminum plate.

Figure 1 depicts an exploded view of the geothermal thermoelectric generator (GTEG) installed at Teide’s fumaroles, whose mode of operation is patented under number WO 2019/202180 A1 [47]. In this view, a cut has been performed in the ground to emphasize the construction and positioning of the hot side heat exchanger, which is in direct contact with the ground in reality. Thus, geothermal heat is absorbed by means of eight 450 mm long grooved tubes made of nickel-plated copper containing water in their interior (Figure 2a). 350 mm of these tubes are in direct contact with the ground, causing the vaporization of the internal fluid, which ascends to the upper part of the pipe, where it condensates releasing heat to the thermoelectric modules. In order to obtain a planar contact surface between the tubes and the thermoelectric modules, the tubes are inserted in semicircular channels milled in a 150 × 90 × 15 mm${}^{3}$ aluminum plate, and pressed afterward, as detailed in Figure 2c.

Two bismuth-telluride thermoelectric modules partially convert the incident heat, which is provided by condensation inside the hot side heat exchanger, into electricity. The remaining heat is released on the other side of the TEG by the cold side heat exchanger. The installed modules are one Marlow TG12-8-01L and one Marlow TG12-8-01LS [48]. The only difference between them is that the latter is sealed with silicone for protection. Furthermore, as shown in Figure 1, a 40 × 40 × 10 $mm^{3}$ aluminum heat extender was added between the hot side heat exchanger and each thermoelectric module since a slight separation of the heat exchangers reduces thermal losses due to thermal bridges [49].

The heat released by the thermoelectric modules is transmitted to the cold side heat exchanger, which is similar to the hot side one, except for the inclusion of 62 aluminum fins with a distance of 5 mm (Figure 2b). In this case, vaporization takes place in the lower part of the heat exchanger, in contact with the thermoelectric modules. The vapor ascends and condensates in the finned part of the tube. Since these fins allow increasing the exchange area with the windy environment, thus its thermal resistance decreases.

**Figure 3:**
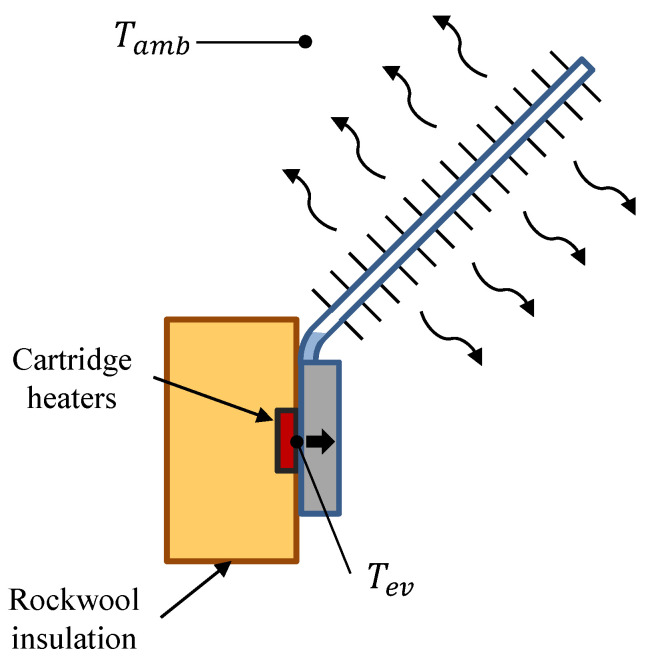
Schematics of the characterization of the cold side heat exchanger.

This cold side heat exchanger has been characterized in order to determine its thermal resistance with respect to the heat flux for different environmental conditions. For this purpose, as shown in Figure 3, cartridge heaters embedded in two 40 × 40 mm${}^{2}$ copper blocks have been used as heat source, simulating the heat released by the thermoelectric modules. In order to ensure that all the heat provided by the power supply goes through the heat exchanger, it has been necessary to add rockwool insulation so that thermal losses into the environment are minimized.

In the experiments, it has been studied the influence of different heat fluxes (75, 100 and 125 W per block) and environmental conditions (pure natural convection as well as 1.6 and 2.9 m/s wind velocities reproduced with a fan). In each case, the thermal resistance per thermoelectric module has been calculated with Equation (1), in which $T_{ev}$ is the temperature measured at the base of the evaporator, $T_{amb}$ is the ambient temperature and $\dot{Q}$ is the useful heat flux per block provided by the power supply, which is in turn calculated as the subtraction of the power (V×I) minus the estimated thermal losses ${\dot{Q}}_{losses}$, for which the estimation of the convective heat transfer coefficient is necessary similarly to [30]. Each experiment has been repeated three times and the uncertainties have been calculated according to [50], leading to an average uncertainty of 4.3%.

(1) $$R=\frac{T_{ev}-T_{amb}}{\dot{Q}}=\frac{T_{ev}-T_{amb}}{V\times I-{\dot{Q}}_{losses}}=\frac{T_{ev}-T_{amb}}{V\times I-h_{ins}\times A_{ins}\times \left(T_{ins}-T_{amb}\right)}$$

**Figure 4:**
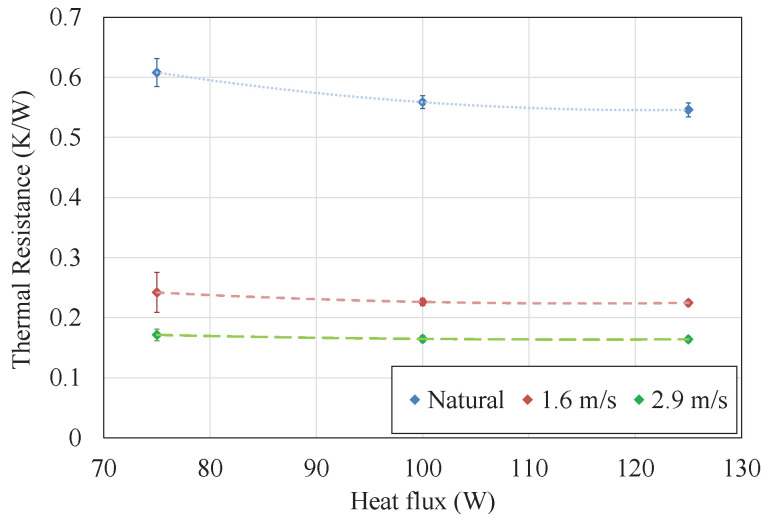
Thermal resistance per thermoelectric module of the cold side heat exchanger for different external conditions. Each experiment has been repeated three times and the uncertainties have been calculated according to [50].

Figure 4 depicts the results of thermal resistance obtained for the different heat fluxes and environmental conditions studied. As can be observed, in all cases the thermal resistance decreases with increasing heat fluxes. This is the conventional behavior of phase change heat exchangers, since the properties of their internal working fluid improve with temperature, leading to a lower thermal resistance. Nonetheless, the biggest influence on the thermal resistance is caused by the exterior conditions, decreasing with higher wind velocities. In forced convection, thermal resistance is practically constant, presenting values of 0.22 and 0.18 K/W with wind velocities of 1.6 and 2.9 m/s respectively. In contrast, in natural convection a more pronounced dependency with respect to the heat flux can be observed, decreasing from a thermal resistance of 0.61 K/W with 75 W to 0.55 K/W with 125 W. The thermal resistance of the cold side heat exchanger can be divided into all the processes that occur within it: Conduction in the lower part of the tubes, boiling, condensation, conduction in the upper part of the tubes and the fins, and convection.

(2) $$R=R_{k1}+R_{b}+R_{cond}+R_{k2}+R_{conv}=\frac{ln(D_{e}/D_{i})}{2\pi Lk}+\frac{1}{h_{b}A_{b}}+\frac{1}{h_{cond}A_{cond}}+\frac{ln(D_{e}/D_{i})}{2\pi Lk}+\frac{1}{h_{conv}A_{conv}\eta_{fins}}$$

It is the latter the one that is influenced by wind velocity. As derived from the Nusselt expressions, in forced convection the convective coefficient $h_{conv}$ depends on Reynolds and Prandtl numbers exclusively and therefore, this coefficient mainly depends on the air velocity, while in natural convection Grashof number also has influence [51]. As shown in Equation (3), Grashof number is directly proportional to the gravity *g*, the coefficient of thermal expansion β that equals to 1/T for ideal gases, the temperature difference between the surface and the ambient $T_{s}-T_{amb}$ and the cube of the characteristic length, and inversely proportional to the square of the kinematic viscosity ν. Hence, if the temperature difference between the external part of the tube and the ambient increases, so does Grashof number, resulting in a greater Nusselt number and consequently a better convective heat transfer coefficient that causes a lower convective thermal resistance. In the experiments, when the heat flux increases, the temperature difference between the surface of the tubes and the heat sink also increases, leading to a lower convective thermal resistance and consequently, to a smaller total thermal resistance of the cold side heat exchanger. Nonetheless, Teide, due to its altitude, is generally windy, so forced convection conditions will be predominant and the heat exchanger’s thermal resistance is expected to be lower than 0.3 K/W.

(3) $$Gr=\frac{g\times \beta \times \left(T_{s}-T_{amb}\right)\times l^{3}}{\nu^{2}}$$

**Figure 5:**
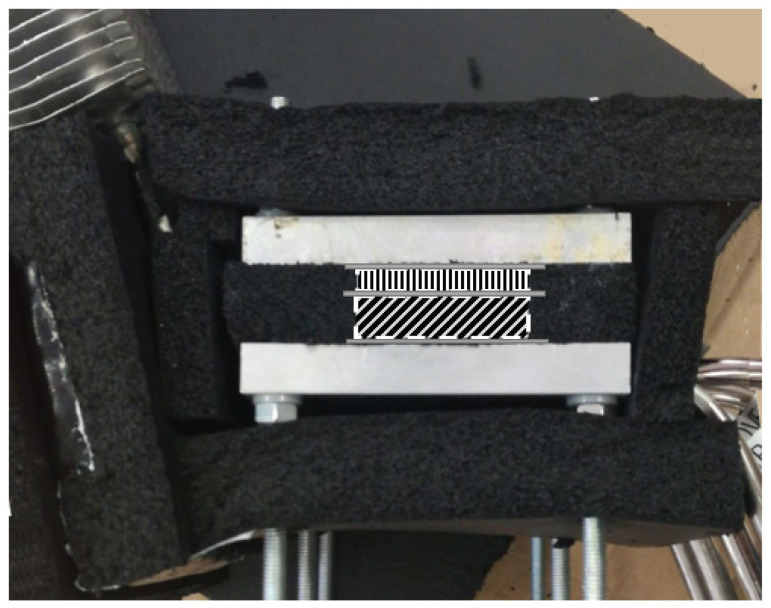
Detail of the neoprene layers installed covering the aluminum plates. On the figure, a drawing of the position of the heat extenders (with diagonal lines), the thermoelectric modules (with vertical lines) and the graphite sheets (in filled gray) has also been added.

All the aforementioned components were assembled by means of six M6 threaded rods that permit holding the prototype in the ground and provide stability. For this purpose, the heat pipe tubes were bent an angle of 69${}^{\circ }$ with respect to the vertical. In order to improve the thermal contact between the thermoelectric modules and the heat exchangers, Panasonic pyrolytic graphite sheets 0.1 mm thick were included [52]. Finally, neoprene layers (10 and 15 mm thick) covered all the exposed parts of the aluminum plates, forcing condensation and vaporization of the hot and cold side heat exchangers respectively, to occur on the thermoelectric modules (Figure 5). This is especially important in the hot side heat exchanger, since it is desirable that all the absorbed geothermal heat goes through the thermoelectric modules, and it is not lost before its transformation into electricity. While the neoprene cover the thermoelectric modules, their position, as well as the heat extenders and graphite sheets one, has been detailed in Figure 5.

**Figure 6:**
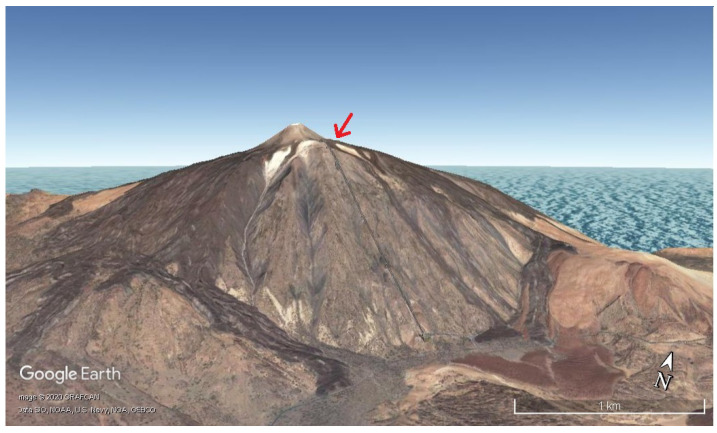
Location of the installed prototype closed to “La Fortaleza” lookout. *©* Google Earth.

**Figure 7:**
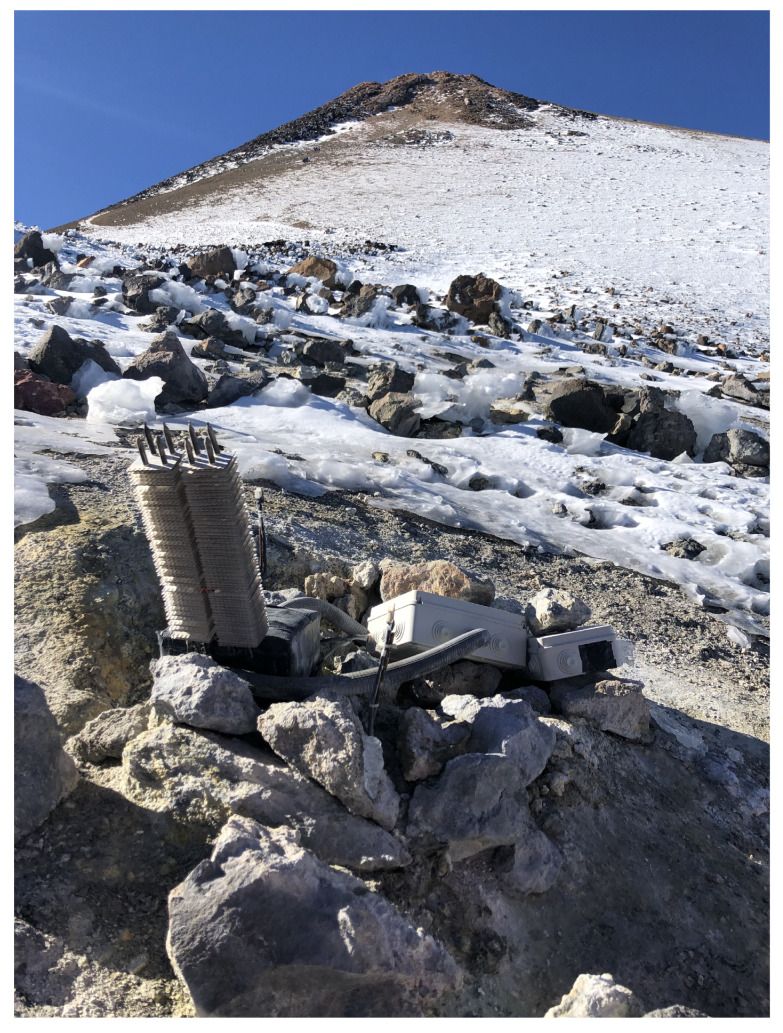
Prototype installed at Teide volcano.

The prototype was installed on 15th March 2019 at Teide volcano, the most emblematic volcano at the Canary Islands (Spain). In particular, it was installed closed to “La Fortaleza” lookout, located at an altitude of approximately 3500 m, facing the northern part of the island (Figure 6 and Figure 7). In this location, there exist fumaroles with a temperature of 82 ${}^{\circ }C$ [31,32].

## 3. Monitoring System

In order to study the viability of thermoelectric generators in volcanic fumaroles, monitoring the prototype installed at Teide volcano becomes indispensable. Hence, 18 thermocouples, four humidity sensors, and two power sensors have been installed.

**Figure 8:**
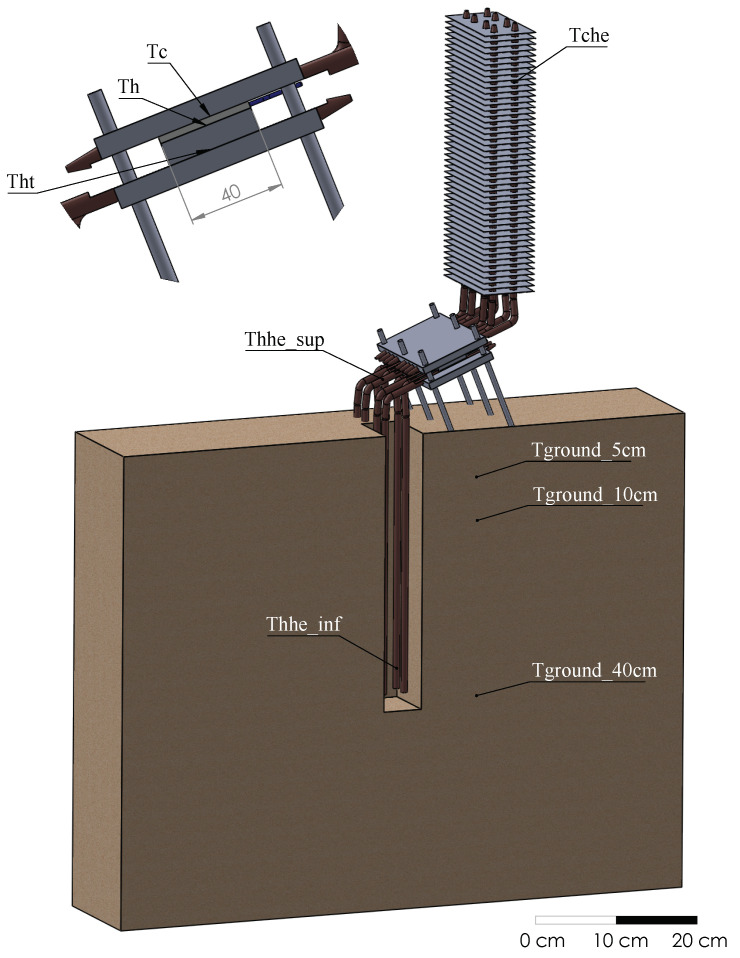
Positioning of the K-type thermocouples.

Figure 8 details the position of the thermocouples, most of which have been duplicated: $T_{ground\_40cm0}$ and $T_{ground\_40cm1}$ are buried in the ground, at an approximate depth of 40 cm; $T_{ground\_10cm}$ and $T_{ground\_5cm}$ are also buried in the ground, but at 10 cm and 5 cm deep respectively; $T_{hhe\_inf0}$ and $T_{hhe\_inf1}$ measure two of the tubes (one corresponding to each module) of the hot side heat exchanger in their lower part, at an approximate depth of 35 cm (neoprene isolation avoids the influence of ground temperature); $T_{hhe\_sup0}$ and $T_{hhe\_sup1}$ are located on the same tubes than the latter, but in its superior part, out of the ground, close to the aluminum plate, and are also protected with neoprene; $T_{ht0}$ and $T_{ht1}$ represent the temperature of the aluminum plate of the hot side heat exchanger, measured thanks to the grooves that can be appreciated in Figure 2c; $T_{h0}$ is the hot side temperature of the Marlow TG12-8-01L thermoelectric module, while $T_{h1}$ refers to the sealed Marlow TG12-8-01LS module; $T_{c0}$ and $T_{c1}$ are the cold side temperature of the thermoelectric modules, measured in the grooves of the cold side aluminum plate; and $T_{che0}$ and $T_{che1}$ indicate the temperature of two tubes of the cold side heat exchanger in the upper part (again each tube corresponds to one module and neoprene isolation has been added).

All the previous temperatures have been measured by means of K-type thermocouples with epoxy coated tips and ± 2 ${}^{\circ }C$ accuracy. Each thermocouple was connected to a MAX31855, an Adafruit breakout board responsible for the amplification of the thermocouples’ signal with cold compensation reference [53]. For protection, all the thermocouples were coated with heat shrink tubing and those buried in the ground were also inserted in a 2 mm brass tube.

In addition, two shielded DTH22 sensors [54] measured the ambient temperature ($T_{amb0}$ and $T_{amb1}$) as well as the humidity ($Hum_{amb0}$ and $Hum_{amb1}$). Ground humidity was also measured by means of two soil moisture sensors buried at a depth of 40 cm [55].

The previous sensors were used in order to monitor the conditions at which the installed thermoelectric generator was working. Nonetheless, it is of utmost importance to determine the power generation of the thermoelectric modules. For this purpose, the thermoelectric modules were individually connected to a 3.2 Ω load resistance as a first approximation. The objective of the present paper is to study the viability of thermoelectric generation from fumaroles, and therefore, a constant load resistance has been connected and its generation has been measured with Adrafuit INA219 breakout boards, with 1% precision [56]. However, in case the viability is demonstrated and these devices are used for a real application, maximum power point trackers will be required, with their associated efficiency that will slightly reduce total generation. Actual MPPTs achieve efficiencies higher than 85% even in ultralow-power applications [57,58].

**Figure 9:**
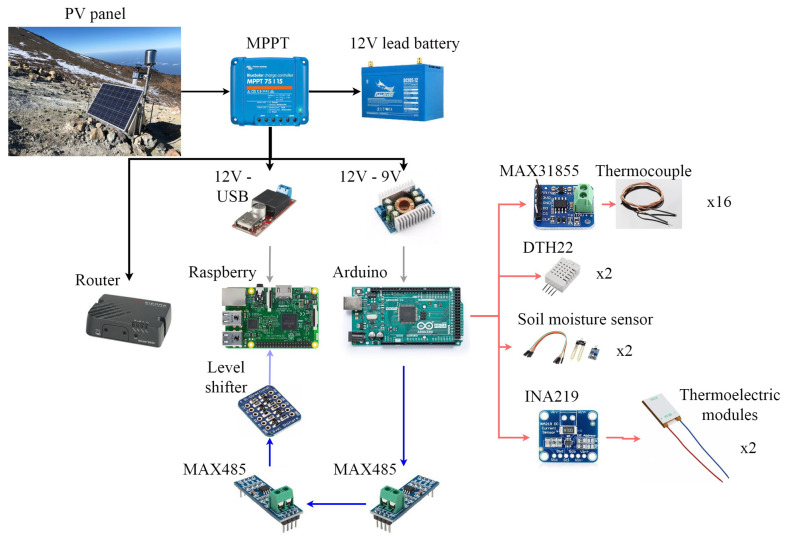
Diagram of the monitoring system including the power supply (black), the RS485 communication (blue), and the sensors (red).

All the temperature, humidity, and power generation sensors were connected to an Arduino Mega 2560, which did a measurement scan every 10 s. This Arduino formatted the measured values into a JSON structure that was sent by RS485 protocol to a Raspberry PI 3 Model B+. RS485 protocol was chosen because the distance between the Arduino and the Raspberry was greater than 5 m, more than the maximum distance supported by USB. The Raspberry stored the received data into InfluxDB, a time series database. This Raspberry was in turn connected to a GSM Router so that its database was synchronized with a private server through MQTT protocol, allowing to see the info in a Grafana dashboard. Figure 9 details the communication between the Arduino and the Raspberry, including the MAX485 converter configured as transmitter in the Arduino and as receptor in the Raspberry [59], as well as the BSS138 logic level converter to adapt the received signal to Raspberry’s GPIO requirements [60]. The schematics also shows the PV panel, including its MPPT and storage system, that was part of the existing volcanic vigilance station located at “La Fortaleza” lookout, and that supplied power to the Arduino, the Raspberry and the GSM router.

In order to protect the electronics from ambient conditions, all the circuits were placed in plastic boxes.

## 4. Results and Discussion

Once the prototype and the monitoring system have been described, the present section shows the results obtained on the 16th and 17th March 2019, after the stabilization of the different variables.

**Figure 10:**
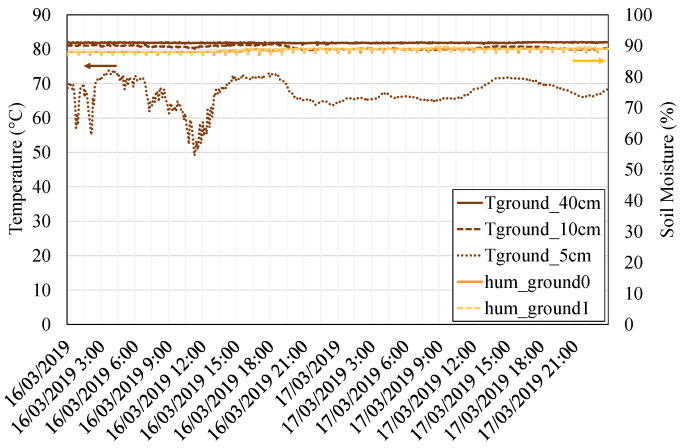
Ground temperature at a depth of 5 cm (dotted brown line), 10 cm (dashed brown line), and 40 cm (filled brown line) on the left axis, and soil moisture at a depth of 40 cm on the right axis (orange and yellow lines).

On the one hand, Figure 10 depicts the conditions of temperature and humidity of the heat source, the fumaroles. The temperature has been measured at a depth of 5, 10, and 40 cm (brown lines). As can be observed, at very low depths, the ground temperature is influenced by ambient conditions. Nonetheless, as depth is increased, ground temperature stabilizes and presents an approximately constant value of 82 ${}^{\circ }C$. Considering that it is necessary to transport geothermal heat from a considerable depth underground to the thermoelectric modules located overground, the selected heat pipes represent an excellent solution since they are capable of transmitting great amounts of heat with a minimum temperature drop as they are based on phase change. Soil moisture, depicted in the right axis of the figure and measured at a depth of 40 cm, also follows a constant tendency, with almost 90% of relative humidity. Since this value is greater than 30%, a good heat transfer from the soil is expected [45].

**Figure 11:**
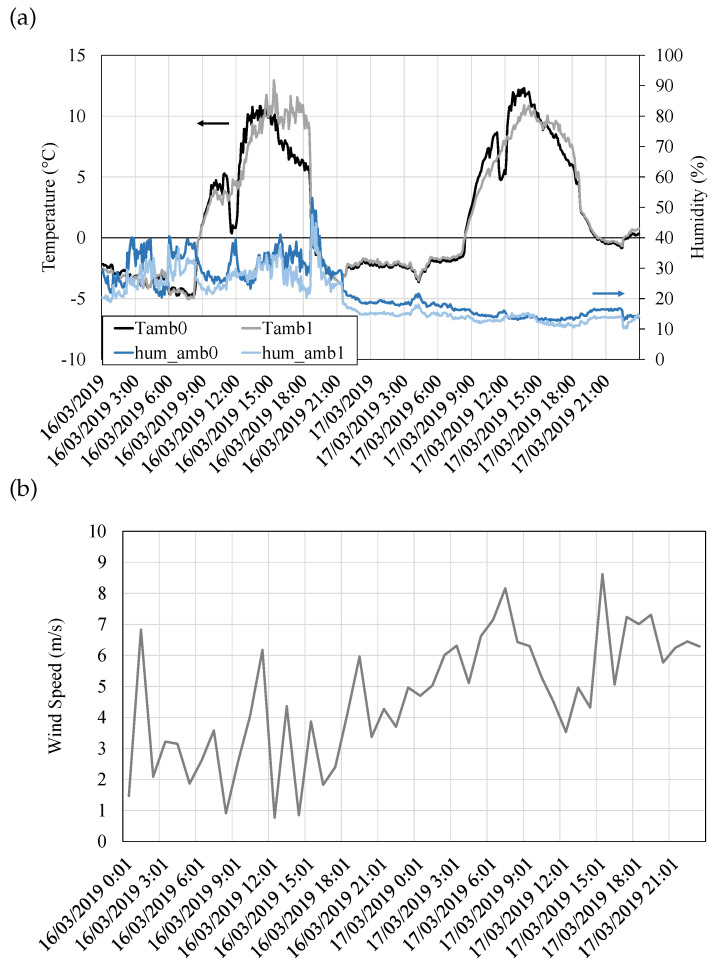
(**a**) Ambient temperature (left axis) and relative humidity (right axis) measured every 10 s. (**b**) Wind velocity measured every hour at a weather station nearby.

On the other hand, the conditions of the heat sink are shown in Figure 11. Ambient temperature (Figure 11a, left axis) varies throughout the day, with temperatures below zero during the night and up to 12 ${}^{\circ }C$ during the day. Slight variations between the sensors are due to their different locations. Figure 11a also depicts the relative ambient humidity in its right axis. Humidity does not follow a clear tendency and differs depending on the considered date. Hence, on 16th March, humidity constantly oscillates between 20 and 50%, while on 17th March, it stabilizes to an approximately constant value of 15%. In order to completely characterize the heat sink conditions, wind velocity has been represented in Figure 11b. These values of wind velocity were obtained from a weather station located nearby, which measured this value every hour. While it would be desirable to have more frequent measurements, the available data shows a typical variant smooth-moderate breeze with wind velocities that oscillate between 1 and 9 m/s, leading to forced convection in the cold side heat exchanger.

The maximum temperature difference achievable between the sides of the thermoelectric modules would equal the subtraction of ground temperature minus ambient temperature ($T_{ground}-T_{amb}$). Nevertheless, the real temperature difference of the thermoelectric modules is always lower. The discrepancy between the real and the ideal values depends on the installed heat exchangers. Thus, the lower the thermal resistance of the heat exchangers, the higher the temperature difference of the thermoelectric modules.

**Figure 12:**
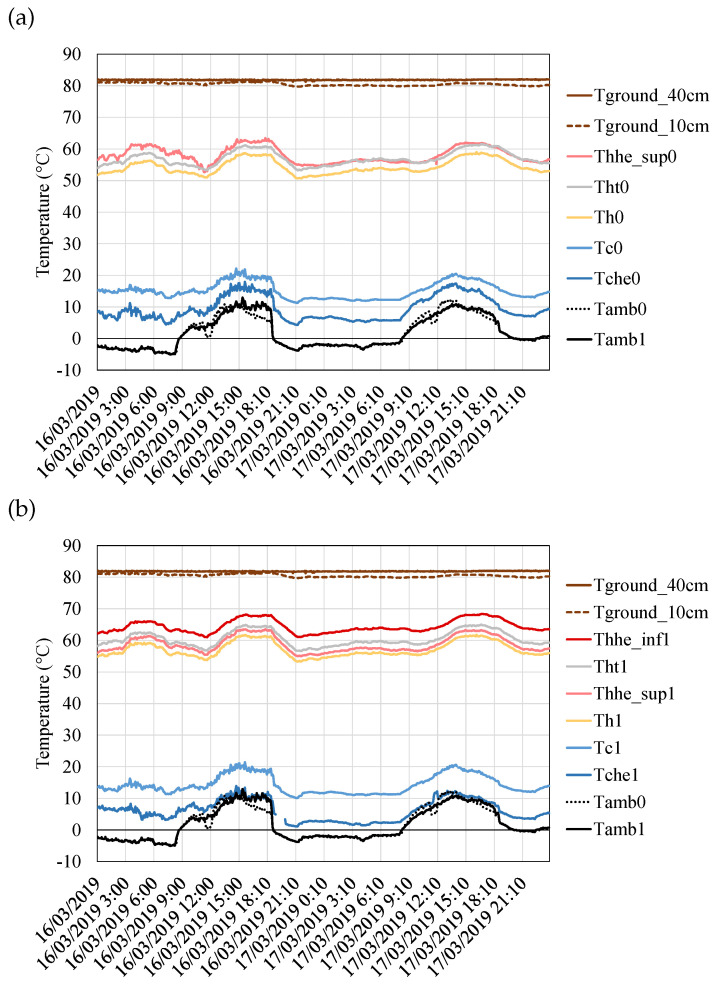
Temperature distribution of the prototype, separated into (**a**) the unsealed thermoelectric module and (**b**) the sealed one. $T_{ground}$ represents the ground temperature measured at depths of 40, 10 and 5 cm; $T_{hhe\_inf}$ and $T_{hhe\_sup}$ are the temperatures in the lower and upper part of the hot side heat exchanger’s tubes respectively; $T_{ht}$ is the temperature of the aluminum plate of the hot side heat exchanger; $T_{h}$ and $T_{c}$ represent the hot and cold side of the modules; $T_{che}$ is the temperature at the tubes of the cold side heat exchanger; and finally $T_{amb}$ measures the ambient temperature.

In this case, the two thermoelectric modules installed, the unsealed and the sealed Marlow TG12-8, have been assembled with the same heat exchangers. Hence, as shown in Figure 12, their temperature difference is similar and it is encompassed in the 36 to 46 ${}^{\circ }C$ range, depending on the ambient conditions. A slightly higher temperature difference can be appreciated in the case of the sealed module ( 3.6 ${}^{\circ }C$ more on average), which is believed to be because of thermal contact and assembly disparities rather than due to the sealant, since the manufacturer states the same behavior regardless of the addition or not of the protection sealant [48].

The effect of the importance of having heat exchangers with low thermal resistance can be also appreciated in Figure 12 comparing the temperature difference between the ground and the hot side of the module ($T_{ground}-T_{h}$) versus the difference between the cold side of the module and the ambient temperature ($T_{c}-T_{amb}$), this is the temperature difference in the hot and the cold side heat pipes. Both heat exchangers had the same structure, but the cold side one included a series of fins. These fins increase the heat transfer area with the environment, which leads to a lower thermal resistance and therefore to a lower temperature difference in the cold side heat exchanger. Hence, the cold side heat pipe has a temperature difference between 8 and 21 ${}^{\circ }C$ while the hot side one presents a difference in the 15 to 27 ${}^{\circ }C$ range.

Apart from the ground, the ambient as well as the modules’ hot and cold side temperatures, more thermocouples have been installed in order to monitor the temperature at other interesting points. In the case of the cold side heat exchanger, $T_{che}$ measured the temperature on the surface of two of the heat pipe tubes (one corresponding to each module), in the upper finned part. As can be seen in Figure 12, these temperatures are approximately in the middle of $T_{che}$ and $T_{amb}$, showing that the convective component of the cold side heat pipe has the same weight in the thermal resistance that all the resting processes (conduction, boiling, and condensation).

In the case of the hot side heat exchanger, two tubes were selected (again one per each module) and thermocouples were located in their lower and upper parts, at a depth of 35 cm in the ground ($T_{hhe-inf}$) and in the bent part respectively ($T_{hhe-sup}$). In addition, two thermocouples measured the temperature $T_{ht}$ at the base of this heat exchanger, before the heat extender introduced between the heat exchanger and the thermoelectric modules. These temperatures are also depicted in Figure 12. Firstly, it can be observed that the introduction of the heat extender causes a slight temperature loss in the hot side of the thermoelectric module, which is quantified at an average of 3 ${}^{\circ }C$. In the case of not introducing it, the temperature of the hot side of the thermoelectric modules would slightly increase, but heat losses through thermal bridges will be higher, reducing total efficiency [49].

Secondly, regarding the temperatures of the tubes, when comparing the thermocouples corresponding to each module, it can be seen that the temperature of the upper part $T_{hhe-sup}$ is similar in both cases, with a tendency clearly affected by the ambient conditions and really close to $T_{ht}$. In the lower part, only $T_{hhe-inf-1}$ could be registered. This temperature is again influenced by the ambient conditions, and it is quite close to $T_{hhe-sup}$. Hence, heat transfer with the ground is the most critical component of the thermal resistance of the hot side heat exchanger. An increase of area, including vertical fins, would improve this heat transfer, leading to a lower thermal resistance and therefore an increase of the temperature difference of the modules and their generation.

**Figure 13:**
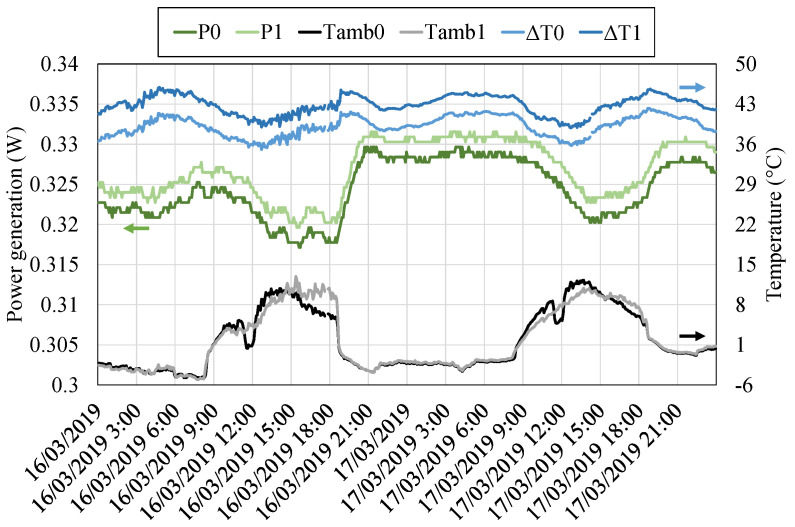
Power generated by the thermoelectric modules (left axis) and ambient temperature (right axis).

Once the temperature distribution has been analyzed, Figure 13 (left axis) shows the generation of the two thermoelectric modules, being $P_{0}$ the power generated by the unsealed module and $P_{1}$ the power corresponding to the sealed one. The sealed module had a greater temperature difference between its sides that translates into a slightly higher generation in comparison with the unsealed one. Apart from this slight difference, both modules follow a similar generation tendency, increasing their generation during the night and decreasing it during the day. In order to have a better perception of this fact, the ambient temperature and the temperature difference accross both modules ΔT have been also represented in the right axis of Figure 13. During the night, ambient temperature decreases and therefore, the temperature difference of the modules increases, leading to a higher generation, which in the sealed module reached up to 0.33 W while in the unsealed one, 0.326 W. During the day, the temperature difference decreases, and so does the generation, with values of around 0.32 W. This effect occurs with a small delay due to the thermal inertia of the different components. Furthermore, it can be also observed that a lower temperature does not necessarily imply a greater generation. During the night of 16th March, the ambient temperature was lower than on the 17th of March. However, generation on the latter is greater due to the higher wind velocity, which improves the convection of the cold side heat exchanger, leading to a lower thermal resistance.

While the generation values could seem scarce, the obtained results are considered of great interest, since this generation can be used to supply power to volcanic monitoring stations, making them completely autonomous. As stated in the introduction, the power requirement of these stations is of just a few watts, and in some cases even of only milliwatts. The latter is the case of Awadallah et al., who required a power consumption of 0.34 mW on average [35]. Thus, the prototype developed in this paper would generate 1000 times more power than required, permitting the installation of more sensors. In those cases that present a higher consumption, one of the main advantages of the proposed device is that, due to the utilization of a constant heat source, the capacity of the required batteries can be greatly reduced, something really interesting since the installed batteries usually have really high capacities [61]. Moreover, the device is very compact and uses passive heat exchangers, reducing maintenance to a minimum due to the absence of mobile parts, aspects of great importance in the application under consideration. Its cost is neither an issue, as thermoelectric generators have demonstrated to be competitive in comparison with other technologies [62].

**Figure 14:**
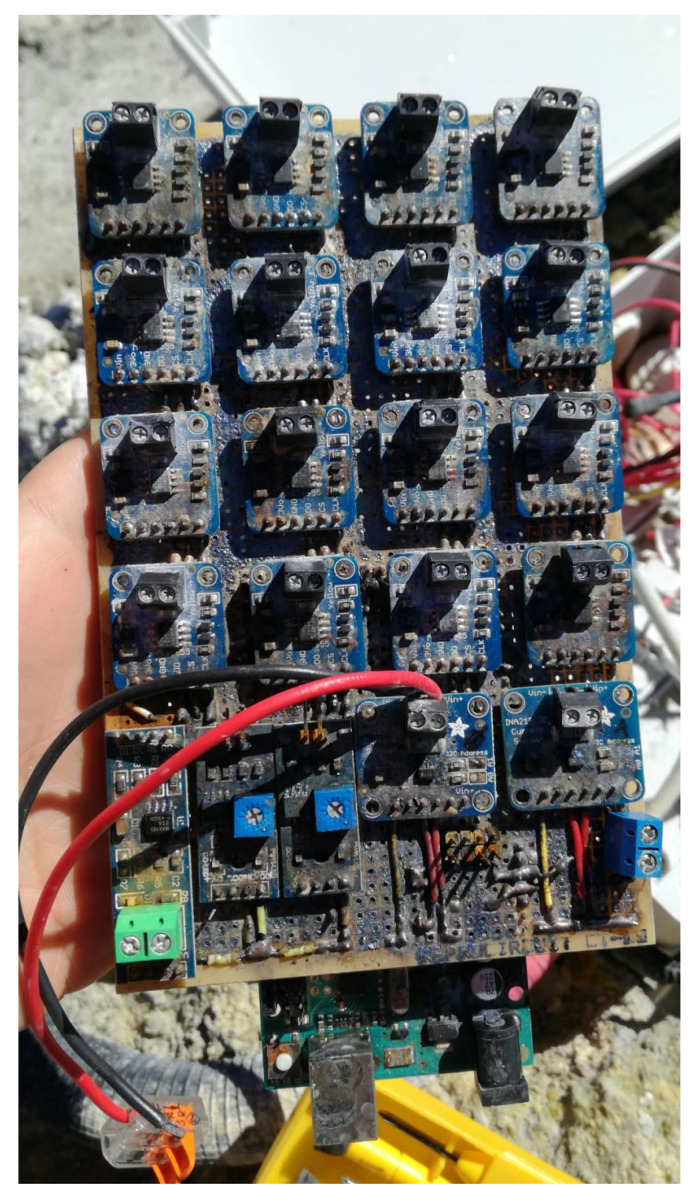
Corroded Printed Circuit Board (PCB) after one month of operation under volcanic conditions at Teide.

In order to fully demonstrate its viability, measures against corrosion need to considered. The monitoring of the different variables stopped after three days, on 19 March 2019. Three weeks later it was possible to examine the prototype, and it was discovered that corrosion had severely affected the electronics, as shown in Figure 14. Volcanic fumaroles present a composition of gases that includes hydrogen sulfide (H${}_{2}$S). Water vapor reacts with hydrogen sulfide, leading to sulfuric acid, which highly corrodes metals, especially copper [63]. The plastic boxes where the electronics was introduced were not sealed, permitting the entry of gases and humidity, and causing corrosion.

**Figure 15:**
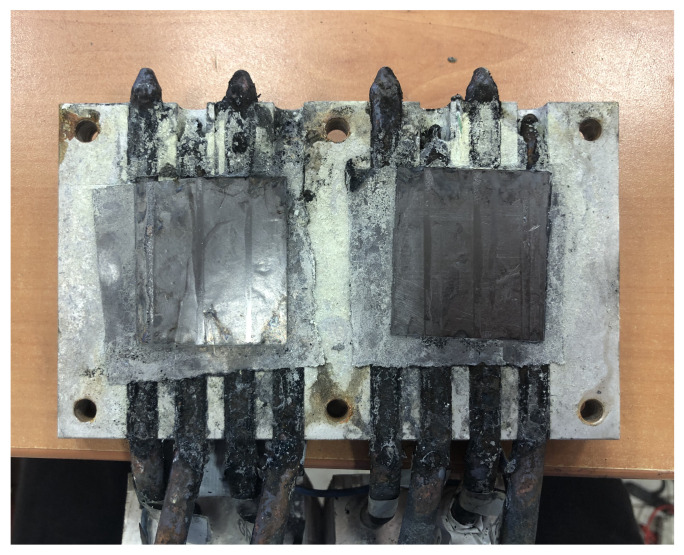
Corrosion of the cold side heat exchanger.

The heat pipe tubes that compose the prototype were also made of copper (nickel-plated), and therefore, signs of corrosion were also perceptible. Figure 15 shows the aluminum plate of the cold side heat exchanger after approximately one month of exposure to Teide’s volcanic environment. As can be observed, excepting the graphite sheet where the thermoelectric modules were placed, all the surface is covered by yellowish deposits of sulfur, despite the fact that it was protected with neoprene and adhesive tape. Hence, in order to achieve the objective of autonomous volcanic monitoring stations, it is important to take measures against the corrosion, protecting better the electronics with a protection of at least IP64 [64], as well as using heat pipes made of more resistant materials such as steel or titanium [65,66], or with protective coatings [67], so that they can resist better in this acidic environment.

## 5. Conclusions

In conclusion, the present paper has experimentally demonstrated, for the first time, the feasibility of thermoelectric generators to generate electricity from fumaroles taking as reference Teide volcano (Canary Islands, Spain), which present 82 ${}^{\circ }C$ fumaroles. The installed thermoelectric generator is capable of generating between 0.32 and 0.33 W per module with a temperature difference between the heat reservoirs of 69 to 86 ${}^{\circ }C$ that includes ambient temperatures below 0 ${}^{\circ }C$. This generation, thanks to the heat pipes used as heat exchangers, based on phase change, is obtained with no auxiliary consumption nor moving parts, leading to a robust generator. This result is especially interesting because the produced electricity could serve in order to supply energy to the volcanic monitoring stations that measure the precursors of the eruptions, making them completely autonomous. Nonetheless, for this purpose, it is necessary to protect the generator against the corrosion provoked by hydrogen sulfide reacting with steam and forming sulfuric acid, which virulently attacks metals, especially copper.

## 6. Patents

The mode of operation of the developed thermoelectric generation is patented under number WO 2019/202180 A1 [47]. 

## Abbreviations

The following nomenclature has been used in this manuscript:

*A*	Area (m${}^{2}$)
*D*	Diameter (m)
*g*	Gravity (m/s${}^{2}$)
Gr	Grashof number
*h*	Heat transfer coefficient (W/m${}^{2}$K)
Hum	Relative humidity (%)
*I*	Intensity (A)
*k*	Thermal conductivity (W/mK)
*l*	Characteristic length (m)
*L*	Length (m)
*P*	Power (W)
$\dot{Q}$	Heat flux (W)
*R*	Thermal resistance (K/W)
*T*	Temperature (${}^{\circ }C$)
*V*	Voltage (V)
ΔT	Temperature difference across the thermoelectric modules
$\eta_{fins}$	Efficiency of the fins
ν	Kinematic viscosity (m${}^{2}$/s)

0	Relative to the unsealed Marlow TG12-8-01L thermoelectric module
1	Relative to the sealed Marlow TG12-8-01LS thermoelectric module
amb	Ambient
*b*	Boiling
*c*	Cold side of the thermoelectric module
che	In the finned part of the cold side heat exchanger
cond	Condensation
conv	Convection
*e*	External
ev	Evaporator’s base
ground_40cm	Buried in the ground at a depth of 40 cm
ground_5cm	Buried in the ground at a depth of 5 cm
ground_10cm	Buried in the ground at a depth of 10 cm
*h*	Hot side of the thermoelectric module
hhe_inf	In the lower part of the hot side heat exchanger
hhe_sup	In the upper part of the hot side heat exchanger
ht	In the aluminum plate of the hot side heat exchanger, before the heat extender
*i*	Internal
ins	In the exterior part of the insulation material
*k*	Conductive
losses	Thermal losses
*s*	Superficial
